# Prospective Case Series of Low-Energy Femtosecond Laser-Assisted Cataract Surgery in Pediatric Patients

**DOI:** 10.3390/jcm14072138

**Published:** 2025-03-21

**Authors:** Luc Van Os, Iske De Backer, Michiel Taal, Marie-José Tassignon

**Affiliations:** 1 Department of Ophthalmology, Antwerp University Hospital, B-2650 Edegem, Belgium; 2 Department of Translational Neurosciences, University of Antwerp, B-2610 Antwerp, Belgium; marie-jose.tassignon@uantwerpen.be

**Keywords:** pediatric lens surgery, femtosecond laser, FLACS, safety, enlargement factor

## Abstract

**Background/Objectives**: We report a consecutive prospective case series to obtain prospective safety and performance data of a low-energy femtosecond (FS)-laser for cataract surgery in children and to evaluate the applicability of the Bochum formula for capsulotomy diameter calculation. **Methods**: In pediatric lens surgery with implantation of a bag-in-the-lens intraocular lens (BIL IOL), anterior capsulotomies were performed using the FS-laser. Regression analysis was used to develop an age-dependent correction formula, and the Pearson correlation was used to evaluate the applicability of the Bochum formula. Surgery-related and ocular adverse events (AE) were recorded at 1 day, 1 week, 1 month, and 6 months postoperatively. **Results**: Thirteen eyes of 10 patients were included in the analysis, among them three cases of subluxated lenses. The mean age was 3.10 ± 2.38 years (range: 4 months to 8 years). The linear bivalent regression yielded the following formula: 1.27 − 0.014 × patient age. Age-related formulas, such as the Bochum formula, are required to calculate the programmed capsulotomy diameter. No complications related to the FS-laser or ocular AEs were observed up to six months; in particular, no excessive shrinkage, retinal detachment, or unusual inflammation were identified. **Conclusions**: This study indicates that the use of the low-energy femtosecond laser for anterior capsulotomy in pediatric lens surgery is safe and contributes to improved reliability and feasibility. The confirmed age dependence of the enlargement factor of the capsulotomy diameter might be related to the higher capsular elasticity in pediatric eyes.

## 1. Introduction

Although pediatric cataracts are a rare disease, it is one of the most common causes of blindness in children worldwide [1]. The incidence of congenital cataracts varies between 24.9 to 63.1 per 100,000 births in Western Europe [2,3,4,5]. One large study in Denmark reported an overall cumulative lifetime risk for cataracts before the age of 18 years of 108.4 per 100,000 children [6]. Early surgery is considered essential in the treatment of young children but is challenging for several reasons, including a high incidence and variety of associated comorbidities, the small size of the eyes, and the differing behavior of tissues compared to adult eyes [7]. The rarity and variability of the disease spectrum results in a long learning curve. One of the particular difficulties in pediatric lens surgery is creating an opening in the capsular bag, a capsulorhexis, which creates access to the opaque lens tissue and to later support the intraocular lens (IOL). The lens capsule in children is more elastic than in adults, and the lens is smaller in size and more curved [8]. This makes manual creation of the capsulorhexis more demanding and is associated with enlargement and increased risk of capsule tears [9].

The precise diameter and continuous circularity of the capsulorhexis are crucial to prevent capsular contraction and influence the position of the IOL, and hence stability, of the postoperative refraction [10,11]. Especially when implanting a bag-in-the-lens IOL (BIL IOL; Morcher GmbH, Stuttgart, Germany), the accuracy of the capsulorhexis diameter and the integrity of the rhexis edge are important [12,13,14]. This is because the IOL will be suspended by the anterior capsulorhexis, as well as by an identically sized posterior capsulorhexis. If one of the rhexes is too small, implantation will not be possible without enlarging the capsulorhexes. If they are too large, the BIL IOL will not have enough support by the lens capsule and risks dislocation.

In this context, the use of a femtosecond laser (FS-laser) can contribute to the accuracy of the capsulotomy while decreasing the number of intraocular manipulations [15,16]. Numerous studies of Femtosecond-laser-assisted cataract surgery (FLACS) in adult patients have shown that capsulotomies created by FS-lasers are precise, predictable, reproducible, allow better IOL centration and minimize the occurrence of surgically induced capsule tears [15,17,18,19,20,21,22]. This is especially an advantage in children because of the limited size of the eyes and the need for optimal precision.

Clinical data related to the use of FLACS in children is limited [23,24,25]. Despite the paucity of available data, femtosecond lasers have been used off-label in cataract surgery in children. Since 2020, the Femto LDV Z8 femtosecond laser has been the only FS-laser system approved for capsulotomy and laser phacofragmentation in pediatric cataract surgery. It is also the only laser used in cataract surgery using low pulse energies in the nanojoule (nJ) range [19,26].

The aim of this study is to obtain prospective data on the safety and performance of the mobile, low-energy FS-laser Femto LDV Z8 for cataract surgery in children and to evaluate the applicability of the Bochum formula for capsulotomy diameter calculation, also in young children.

## 2. Materials and Methods

This consecutive prospective case series was conducted at the Ophthalmology Department of the Antwerp University Hospital, Edegem, Belgium (NCT05241756), in accordance with the tenets of the Declaration of Helsinki. Ethical approval was obtained from the Ethics Committee of the University of Antwerpen (Edge 001299; 17 January 2022). Written informed consent was obtained from the patient’s legal representatives and from the child when aged >6 years old.

The study recruited and consecutively operated on children under the age of 18 who underwent lens surgery between January and July 2022. Criteria for exclusion were eyes with a corneal diameter < 9 mm, a hazy cornea, or pregnancy. All children underwent preoperative and postoperative ophthalmological examinations, including anterior segment biomicroscopy, refraction, eye fundus evaluation, and biometry (either optical biometry or a combination of keratometry and A-scan ultrasound in younger children). In case of insufficient cooperation, the ophthalmological examination was performed under general anesthesia directly prior to the surgery but during the same surgical procedure.

All surgeries were performed by one of two experienced pediatric cataract surgeons (L.V.O. and M.J.T.). The surgery was performed under general anesthesia with constant supervision of an anesthesiologist. After successful docking of the surgical equipment to the patient’s eye, anterior capsulotomy was performed with the FS-laser system, followed by complete aspiration of the contents of the lens, manual posterior capsulorhexis, and implantation of a BIL IOL. Apart from the laser capsulotomy and the verification of the capsulotomy size, the surgeries followed the standard pediatric BIL protocol [14].

The programmed capsulotomy diameter was between 2.3 and 2.9 mm. The calculation was based on the targeted capsulotomy diameter and the Bochum formula [24] to account for the enlargement of the capsulotomy in children due to the high elasticity of the lens capsule. The achieved capsulotomy diameter was measured intraoperatively using the Morcher ring caliper Type 4L (Morcher, Stuttgart, Germany). This ring-shaped device with an internal diameter of 4.5 mm was inserted into the eye after the creation of the capsulotomy and after injecting an ophthalmic viscosurgical device into the anterior chamber. This provides an immediate clinical impression of the obtained size of the capsulotomy. To calculate the true size of the achieved capsulotomy, we extracted a still from the video documentation of the surgeries. Knowing the size of the caliper enabled us to calculate the achieved capsulotomy size using a rule of three. This method thus avoids any influence of the corneal curvature on the measurement of the capsulotomy size.

In order to assess the safety of the procedure (the study’s primary objective), any adverse event (AE) related to the surgery or any ophthalmic AE at 1 day, 1 week, 1 month, and 6 months postoperatively was documented.

The analyzed data set comprised all eyes with successful docking of the FS-laser. All statistical analyses were exploratory, and there were no predefined hypotheses. Statistical analysis was performed using Microsoft Excel 365 for data entry, organization, and structuring and the Data Analysis ToolPak for descriptive statistics and regression analysis. Regression analysis was used to describe the extent to which the capsulotomy enlargement factor was age-dependent. To test the correlation between the patient’s age and the capsulotomy enlargement factor, the Pearson correlation coefficient (r) was used. A *p*-value < 0.001 was considered statistically significant.

## 3. Results

The mean age of patients in the analysis set was 3.10 ± 2.38 years, and the median age was 4 years (range 4 months to 8 years). Table 1 shows the demographic data and comorbidities of the patients, as well as eye characteristics.

**Figure 1:**
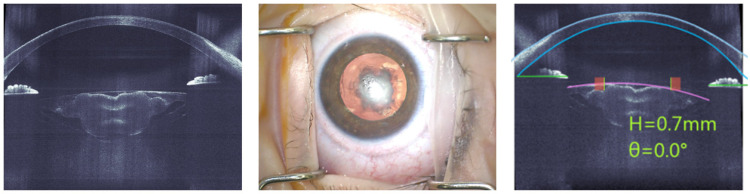
Case of a 7-month-old child with unilateral cataract, central lens opacity, and large posterior plaque overlying a pre-existing posterior capsule defect in a context of anterior vitreolenticular interface dysgenesis. Intraoperative microscope image (**middle**) and high-resolution optical coherence spectral domain tomography (SD-OCT) of the FS-laser (**left**), showing the central location of the lens opacity and its inhomogeneous appearance. The overlay of the FS-laser (**right**) shows automatically identified edges of the cornea and lens capsule, the planned capsulotomy diameter, and the proposed position in the center of the pupil.

We would like to highlight that because we are a referral center for pediatric cataracts, most of the children did present some degree of divergency in their ocular parameters. Due to the small lid opening of the very young children’s eyes, very flat or steep cornea, and the presence of a bleb after glaucoma surgery, docking issues with the standard disposable attachment interface occurred in eight eyes of seven patients (in median 1.17 years; range 4 months to 17 years), and surgery was successfully completed manually. In all 13 eyes (10 patients) with completed docking, the anterior segment was immediately successfully imaged by the high-resolution spectral-domain optical coherence tomography (SD-OCT) of the FS-laser. The margins of the cornea and the lens capsule were automatically identified and displayed together with the planned capsulotomy diameter and the suggested position at the center of the pupil (Figure 1). Manual adjustments were not necessary in any case. A well-centered and entirely circular anterior capsulotomy was successfully performed with the FS-laser in all eyes of the analysis set. Figure 1 shows the case of a 7-month-old child with unilateral cataract, central lens opacity, and large posterior plaque, which was overlying a pre-existing posterior capsule defect in the context of an anterior vitreolenticular interface dysgenesis. The OCT image shows the central location and inhomogeneity of the lens opacity. As the capsulotomy was created with the FS-Laser, the anterior capsule did not require staining, which would have been necessary with a manual capsulorhexis.

**Figure 2:**
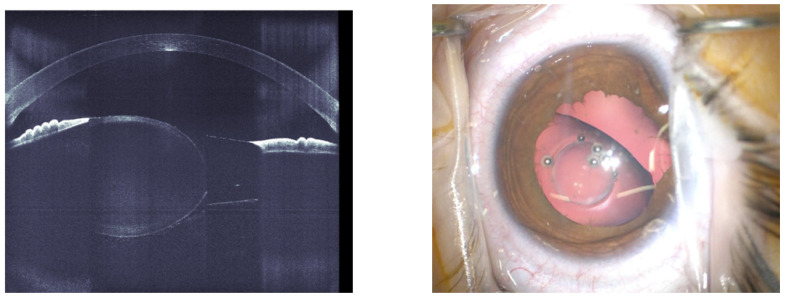
Case of 6-year-old child with subluxated lens. OCT (**left**) and intraoperative microscope image (**right**).

Even in the three eyes with lens subluxation, the FS-laser easily allowed for the creation of a capsulotomy. Figure 2 shows the case of a 6-year-old child with unilateral familial lens subluxation. The OCT (left) illustrates the dislocation as well as the increased convexity of the lens due to the weak zonules. The intra-operative microscope image shows the capsulotomy with a small diameter of 3 mm, which was created by the FS-laser. Through this small capsulotomy, the lens material could be removed by irrigation and aspiration. Afterwards, a capsule tension ring was inserted into the capsular bag, and the capsulotomy was manually enlarged to 4.8 mm, which was needed for a BIL implantation. After the creation of an equally sized posterior rhexis, the BIL was positioned. To further improve stability, two bean-shaped ring segments (Morcher GmbH, Stuttgart, Germany) were inserted in the ciliary sulcus, and the peripheral groove of the BIL and centration was enhanced with a Prolene 6/0 Loop fixed to the sclera. The technique has already been described in detail [27]. In one eye with anterior capsule fibrosis, minimal tissue bridges were resolved manually with microforceps. In three eyes, a small anterior capsular tear developed during the positioning of the BIL IOL; however, IOL implantation was successfully completed in all cases, with a stable IOL position at the end of the surgery. No clinically relevant FS-laser-related complications or ocular AEs were observed up to the end of the 6-month follow-up period. In particular, excessive shrinkage of the capsule, retinal detachment or unusual inflammation were not identified. All eyes were successfully implanted with a BIL IOL.

**Figure 3:**
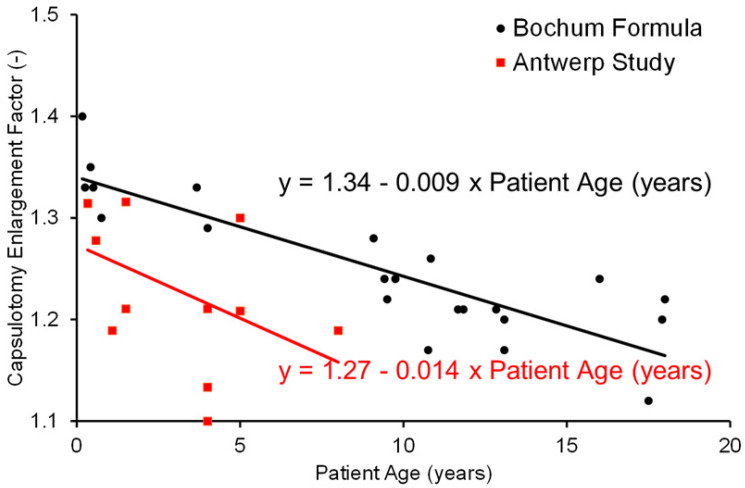
Relationship between capsulotomy enlargement factor and age in FS-laser assisted cataract surgery in children and comparison with data from the study by Dick et al. [18].

Immediately after FS-laser treatment, all eyes showed symmetrical enlargement of the anterior capsulotomy. The linear bivalent regression resulted in the following formula in our analysis set: 1.27 − 0.014 × patient age. Figure 3 shows the enlargement factor in relation to patient age in this study compared to the data of the study by Dick et al. [24]. The Bochum formula calculates the enlargement factor as follows: capsulotomy enlargement factor = 1.34 + (−0.009 × age in years). On the other hand, Liao’s formula calculates the enlargement index thus: predicted capsulorhexis enlargement index = 1.177 − 0.052 × anterior chamber depth + 0.009 × axial length.

Both data sets are normally distributed (tested with Kolmogorov–Smirnov test for normality-*p*-value 0.192 and 0.227 for Liao and Bochum enlargement, respectively). A *t*-test on the differences between the age-dependent changes in enlargement factors for the Antwerp study compared to the Bochum formula demonstrates that the differences are statistically significant (*p* < 0.05). The large variability in our data associated with the calculated age-dependent capsulotomy enlargement factor is clearly visible in Figure 3 and is demonstrated by the weak r2 coefficient of 0.2356.

Calculation of the Pearson correlation was used to assess the applicability of the age-dependence of the Bochum formula for calculating the capsulotomy diameter to be entered into the Femto LDV Z8 (Table 2) [24].

Other formulas have been proposed, taking into account measured physical properties of the eye to calculate the enlargement factor. Liao et al. [28] proposed a relationship between the anterior chamber depth and the axial length to predict the capsulorhexis enlargement factor. While there was only a very weak correlation between the patient’s age or physical properties of the eye and the enlargement factor of Liao et al. [28], the analysis of the new data from this study confirmed the strong correlation between age and the enlargement factor of the Bochum formula suggested by Dick et al., 2015. [24] The lower r2 coefficient we found for the Liao enlargement factor compared to the Bochum formula demonstrates that the physiological parameters of the anterior chamber depth and axial length have a lower correlation to the enlargement factor than considering purely the patient age, especially in the smaller age range on which the Liao formula is derived compared to the Bochum formula, which included children over a much larger age range.

## 4. Discussion

This study represents the first prospective analysis of FLACS in children utilizing the sole FS-laser system approved for this purpose, the Femto LDV Z8. Our findings demonstrate that using this low-energy FS-laser to create the anterior capsulotomy for lens surgery in children is safe. In all cases, a precise, accurately positioned, and perfectly circular capsulotomy was created by the FS-laser after successful docking.

Cataract surgery in children presents a unique set of challenges due to the highly variable anatomical situations and comorbidities, including, amongst others, a fibrotic lens capsule, lens luxation, or a narrow anterior chamber [7]. In these cases, the use of the FS-laser has already been shown to be advantageous in adults and contributed to greater safety in the clinical outcome and precision in the capsulotomy cut [20,21]. This is also confirmed by our case series in children.

In the three cases of lens luxation, a precise capsulotomy was created reliably and with less manipulation using the FS-laser. Manual capsulorhexis in eyes with lens luxation or Marfan syndrome can be a major challenge and carries risks, as unstable zonules and varying vector forces make the manual preparation procedure unpredictable [29]. In these cases, in particular, the use of the FS-laser for capsulotomy can contribute to greater safety and standardization of the clinical procedure. Creating a small capsulotomy with the FS-laser allows for the removal of the lens tissue and subsequent positioning of a capsular tension ring in the capsular bag. This balances the tractional forces to a certain extent and facilitates enlarging the capsulotomy and improving centration.

Another common challenge in pediatric lens surgery is the presence of anterior capsule fibrosis [30], which often makes manual capsulorhexis considerably more difficult and complex. In contrast, in our case of anterior capsule fibrosis, a completely circular and well-centered capsulotomy could be achieved with the FS-laser, and any remaining minimal tissue bridges were removed with minimal effort. In pediatric cataract surgery, staining of the anterior capsule is often necessary due to dense opacities. Using the FS-laser for anterior capsulotomy eliminates the need for staining and its potential negative effects.

In three eyes, slight capsular tears occurred during BIL-IOL implantation, which, however, did not affect the IOL implantation. This might be related to the smaller capsulotomy diameter in combination with the implantation of the BIL-IOL. Due to the enlargement factor given by the elasticity of the lens capsules in young eyes, the actual capsulotomy diameters cut with the laser were consistently smaller than in adults. At the same time, when positioning the BIL-IOL, a certain tension is exerted on the edge of the capsulotomy. This is usually not a critical step due to the high elasticity of the capsule, but it can lead to a capsular tear if there is a small irregularity.

With regard to the calculation of capsulotomy size, our results confirm that there is an enlargement of the capsulotomy compared to the size that has been put into the FS-laser device. Despite the large variability in the data, the trends are consistent with the previously reported findings that younger children exhibit greater capsulotomy enlargement and that an enlargement factor can be used to calculate the targeted capsulotomy diameter. The age dependence of the enlargement factor might be explained by the higher elasticity of the capsule in pediatric eyes [24]. Another factor that might contribute is the maturation of the zonular fibers, which may present individual variations in length and strength and, as a result, may show variations in tensile forces exerted by them on the capsule and, thus, the enlargement effect on the capsulotomy [31].

While the formula of Liao et al., which was determined for children aged 2–6 years based on anatomical parameters, only showed a very weak correlation of the enlargement factor with age (Table 2) [28], the Bochum formula also demonstrated an age-dependent enlargement factor. Accordingly, the use of a formula such as the Bochum formula with an age-dependent enlargement factor appears to be necessary for calculating the achieved capsulotomy diameter for the FEMTO LDV Z8. However, the younger population in this new data sample, as compared to the population analyzed in Bochum [24], does not permit a conclusion of whether the magnitude of the enlargement factor is appropriate over the wider age range reported in the determination of the Bochum formula.

Our results indicate a trend towards a smaller enlargement factor than the Bochum formula, even though a higher enlargement factor could have been expected given the younger population in our study. Several factors could be influencing this, such as the type of cataract, other lens characteristics, or laser parameters that could influence the capsulotomy edge. Furthermore, because of the importance of capsulotomy size in BIL implantation, we preferred to rather end up with a slightly smaller capsulotomy than to risk a capsulotomy that would be too large. Further studies are necessary to substantiate this trend in a larger sample and to determine a more exact enlargement factor that is applicable to the FEMTO LDV Z8.

The Z8 is a femtosecond laser system that, thanks to its mobility and small footprint, enables operations to be performed without having to significantly change operating room workflows in terms of space and equipment so that existing workflows are maintained [19]. The mobility of the Z8 was particularly advantageous for these very young patients under general anesthesia, as the children did not have to be moved for the procedure but could be operated on as usual. Although docking with the standard interface was not possible for some children in this first case series at our clinic, an improved docking rate is expected with increasing surgical experience in using the device. In general, it may be advisable for surgeons to gain initial experience with the FS-laser in adults before applying it to children.

During the 6-month follow-up time within this study, no ocular AEs were registered. There were no cases of increased eye pressure related to the procedure, and there were no cases showing an increased inflammatory postoperative reaction. The absence of postoperative inflammation might be related to the low-energy setting of this type of FS-laser [21,32].

Limitations of this prospective case series include the lack of a control group with manual cataract surgery, the small number of eyes in the data sample size, and the exploratory data analysis without testing an existing hypothesis. Nevertheless, this study provides important prospective data on the safety and efficacy of cataract surgery with the FEMTO LDV Z8 in children. In the future, the possibility of posterior capsulotomy with the low-energy FS-laser could also be investigated.

## 5. Conclusions

FS-laser-assisted pediatric cataract surgery with the low-energy FS laser is a safe procedure with none of the patients in our series suffering ocular AE during a 6 month follow-up period. The FS-laser can simplify the creation of an anterior capsulorhexis in challenging lens surgery in children, especially in cases of white cataracts, lens luxation, and subcapsular fibrosis. The development of an adapted interface for docking could increase the number of children in whom the FS-laser platform can be used, especially for surgery in very young children with small eyelid apertures. We were able to confirm the age dependency of the capsulotomy diameter enlargement factor described in earlier studies [24,28]. The enlargement of the capsulotomy might be related to the higher capsular elasticity and stronger zonular fibers in pediatric eyes. Further research within larger groups of patients is needed to finetune the enlargement factor, which might also vary between different FS-laser platforms.

## Figures and Tables

**Table 1:** Demographics and eye characteristics. (ACD: anterior chamber depth, K1: keratometry along the flattest axis, K2: keratometry along the steepest axis, SD: standard deviation).

Patient characteristics (n = 13)	Value
Age (years), mean ± SD (range)	3.10 ± 2.38 (4 months to 8 years)
Female, n (%)	53.83%
**Eye characteristics (n = 13)**	
Right eye, n (%)	7 (54%)
Lens luxation, n (%)	3 (23%)
Axial length (mm)	21.78 ± 1.98
ACD (mm)	2.85 ± 0.97
White-to-white (mm)	11.26 ± 0.59
K1 (D)	43.00 ± 1.20
K2 (D)	44.49 ± 2.55
Data are presented as mean ± SD, if not indicated differently.	

**Table 2:** The Pearson correlation to assess the applicability of the age dependence of the Bochum and Liao formula for calculating the capsulotomy diameter.

Enlargement Factor	Pearson Coefficient (r)	*p*-Value	Adjusted R-Squared	Sample Size (n)
This study, calculated according to Liao [28]	−0.231	0.447	0.053	13
This study, calculated with the Bochum factor [24]	−0.984	<0.001	0.968	13
Dick et al., 2015 [24]	−0.865	<0.001	0.749	22

## Data Availability

Dataset available upon request from the authors.

## Abbreviations

The following abbreviations are used in this manuscript:

FS-laser	femtosecond laser
BIL	bag-in-the-lens
IOL	intraocular lens
AE	adverse events
FLACS	femtosecond laser-assisted cataract surgery
SD-OCT	spectral-domain optical coherence tomography
